# Haemoglobin and transfusions in elderly patients with hip fractures: the effect of a dedicated orthogeriatrician

**DOI:** 10.1186/s13018-021-02524-0

**Published:** 2021-06-16

**Authors:** Marco Quaranta, Luca Miranda, Francesco Oliva, Filippo Migliorini, Gabriela Pezzuti, Nicola Maffulli

**Affiliations:** 1 Department of Musculoskeletal Disorders, Faculty of Medicine and Surgery, University of Salerno, 84084 Baronissi, Italy; 2 Clinica Ortopedica, Ospedale San Giovanni di Dio e Ruggi D’Aragona, 84131 Salerno, Italy; 3 Department of Orthopedic and Trauma Surgery, RWTH University Clinic Aachen, Pauwelsstr. 31, 52074 Aachen, Germany; 4 Queen Mary University of London, Barts and the London School of Medicine and Dentistry, Centre for Sports and Exercise Medicine, Mile End Hospital, 275 Bancroft Road, London, E1 4DG England; 5 Guy Hilton Research Centre, Faculty of Medicine, School of Pharmacy and Bioengineering, Keele University, Thornburrow Drive, Hartshill, Stoke-on-Trent, ST4 7QB England

**Keywords:** Haemoglobin, Transfusions, Orthogeriatrician, Hip fractures, Elderly patients, Interdisciplinary management

## Abstract

**Background:**

Hip fractures are common in elderly patients, in whom it is important to monitor blood loss; however, unnecessary transfusions should be avoided. The primary objective of this study was to assess whether the employment of a dedicated orthogeriatrician in an Orthopaedic Department allows to optimise the clinical conditions of patients, influencing blood loss and consequently the number of transfusions. The secondary objective was to determine whether the influence of the orthogeriatrician differs according to the type of surgical treatment.

**Methods:**

A total of 620 elderly patients treated for hip fracture were included in the study. These patients were divided into two groups according to the presence or not of the orthogeriatrician. For each patient, age, sex, comorbidities, type of fracture, surgical treatment, length of hospital stay, time from hospitalisation and surgery, time from surgery to discharge, haemoglobin (Hb) values (admission, 24h post-surgery, lowest Hb reached, discharge) and the number of transfusions were recorded.

**Results:**

Regardless of the surgical procedure performed, in patients managed by the orthogeriatrician, the Hb at discharge was significantly higher (*p* = 0.003). In addition to the highest Hb at discharge, in patients who underwent hemiarthroplasty, the number of transfusions per patient is significantly reduced (*p* = 0.03).

**Conclusion:**

The introduction of the orthogeriatrician in an orthopaedic ward for the management of elderly patients treated for hip fracture allows to discharge the patients with higher Hb values, reducing the risk of anemisation and the costs related to possible re-admission.

## Introduction

Hip fractures are common in elderly patients [1–6]. They can be classified as medial, or intracapsular fractures, related to an increased risk of osteonecrosis of the femoral head [7–12] and lateral, or extracapsular fractures [13–16]. The main risk factor for hip fractures is osteoporosis [2, 17–20]. Type II osteoporosis, which is age-related, is a risk factor for both female and male patients [2, 17, 18, 20]; type I, postmenopausal, is responsible for the increased incidence of hip fractures in female patients [21–23]. Most patients report an accidental fall, often the result of a bone failure related to advanced osteoporosis [1, 2, 4, 6]. These patients often have comorbidities, especially cardiovascular [1, 4, 16, 24–26]. In patients with hip fractures, it is important to monitor blood loss caused by both trauma and subsequent surgical treatment. Therefore, it is necessary to constantly examine laboratory tests, such as haematocrit and haemoglobin (Hb), so as to intervene promptly with blood transfusions to restore acceptable values [27–30]. However, unnecessary transfusions should be avoided because they represent both a risk factor for adverse outcomes and unjustified expenditure [27–30]. In this perspective, a close collaboration between orthopaedic and orthogeriatrician may provide both clinical and economic advantages. The orthogeriatrician plays a crucial role enhancing and stabilising medical conditions to allow faster access to the actual surgery and reduce the postoperative length of the hospital stay [2].

The primary objective of the present study is to assess whether, in the interdisciplinary management of elderly patients with hip fracture, the employment of a dedicated orthogeriatrician allows to optimise the clinical conditions of patients, influencing blood loss and consequently the number of transfusions. The secondary objective was to determine whether the influence of the orthogeriatrician differs according to the type of surgical treatment: cephalomedullary nail, hip hemiarthroplasty (HHA) or total hip arthroplasty (THA).

## Materials and methods

### Study design

Data were collected from the Academic Department of Trauma and Orthopaedics of the San Giovanni di Dio e Ruggi d’Aragona Hospital of Salerno database. Data were collected for elderly patients undergoing hip fracture surgery for 2 years. From February 1, 2017, to January 31, 2018, before a dedicated orthogeriatrician started to work in the department; and from February 1, 2018, to January 31, 2019, with the employment of the orthogeriatrician in the department. The inclusion criteria were age ≥ 65, intra- and extra-capsular femoral fractures, fragility fractures and low energy trauma. Patients < 65 years with hip fractures resulting from high-energy trauma and/or pathological fractures were excluded. Considering the inclusion and exclusion criteria, a total of 620 patients were included in the study. These patients were divided into two groups according to the presence or not of the orthogeriatrician: 296 patients were included in the Pre-O group (February 1, 2017–January 31, 2018), and 324 patients in the Post-O group (February 1, 2018–January 31, 2019). Two orthopaedic residents worked on data collection and statistical analysis. For each patient, we recorded age, sex, comorbidities, type of fracture (intra/extra-capsular), surgical treatment performed, length of hospital stay (LOS), time from hospitalisation and surgery, time from surgery to discharge, haemoglobin (Hb) values (admission, 24h post-surgery, lowest Hb reached, discharge) and the number of transfusions.

### Orthopaedic surgeon-orthogeriatrician: coordinated activity

Every day, each patient admitted is evaluated with the close collaboration between the staff of the orthopaedic surgeon and the orthogeriatrician. The orthogeriatrician evaluates and manages the general health conditions of the patients, while the orthopaedic deals with the clinical and surgical aspects of the fracture. Following the evaluation of laboratory parameters, management is reassessed and changed if indicated. The levels of Hb are followed to intervene promptly with transfusions, thus avoiding the risk of anaemia. The threshold values of Hb to start the transfusion are debated; often, the decision to transfuse is related to the clinical conditions of patients and associated comorbidities and not to the actual absolute value of haemoglobin concentration.

### Statistical analysis

Descriptive statistic was used for data related to the demographic characteristics of the two groups (mean, range, standard deviation, and percentages). The statistical analysis of continuous variables was performed using the Student t test, while categorical variables were analysed with the chi-square test. The test is considered statistically significant if the *p* value is < 0.05.

## Results

A total of 620 patients were identified and included in the study; 479 (77.3%) were women and 141 (22.7%) men. The average age was 83.85 years (SD 7.35; range from 53 to 99 years). The Pre-O group includes 296 (47.7%) patients, 71 (23.9%) males and 225 (76.1%) females, with an average age of 84.1 years (SD 7.02; range from 63 to 98). One hundred ninety-eight (66.89%) patients were managed surgically with a cephalomedullary nail; 90 (30.41%) with HHA and the remaining 8 (2.7%) patients with THA. The Post-O group includes 324 (52.26%) patients, 70 (21.6%) males and 254 (78.4%) females; the average age was 83.6 years (SD 7.64; range from 53 to 99). In this group, 210 (64.81%) patients were managed with a cephalomedullary nail, 97 (29.95%) with HHA and 17 (5.24%) with THA.

Table 1 summarises the demographic characteristics of the two groups.

**Table 1:** demographic characteristics of Pre-O and Post-O groups

	Pre-orthogeriatrician	Post-orthogeriatrician	***P*** value
**Number of patients**	296	324	
**Mean age** (SD; range)	84.1 (SD 7.02; 63–98)	83.6 (SD 7.64; 53–99)	0.4
**Sex**	71 M; 225 F	70 M; 254 F	
**Cephalomedullary nail**	198 (66.89%)	210 (64.81%)	0.56
**HHA**	90 (30.41%)	97 (29.95%)	0.9
**THA**	8 (2.70%)	17 (5.24%)	0.1
**Length of hospital stay** (mean days; SD; range)	11.84 (4.34; 5 to 45)	12.29 (4.49; 5 to 39)	0.2
**Comorbidities**			
Diabetes	59 (19.93%)	73 (22.53%)	0.4
Hypertension	193 (65.2%)	213 (65.74%)	0.9
Atrial fibrillation	51 (17.23%)	61 (20.61%)	0.3
Heart failure	37 (12.5%)	20 (6.17%)	0.007 *
Myocardial infarction	21 (7.09%)	28 (8.64%)	0.5
Hypercholesterolaemia	33 (11.15%)	47 (14.51%)	0.2
Depression	14 (4.73%)	21 (6.48%)	0.3
TIA	29 (9.79%)	32 (9.87%)	0.9
COPD	30 (10.13%)	26 (8.02%)	0.4
CKD	25 (8.45%)	34 (10.49%)	0.4
Dementia	36 (12.16%)	39 (12.03%)	0.9
Alzheimer	11 (3.72%)	6 (1.85%)	0.2
Parkinson	12 (4.05%)	18 (5.55%)	0.4

### Hb and transfusions

Analysing the values of Hb for each group, regardless of the surgical procedure performed, we obtained data reported in Table 2. No complications related to the blood transfusions were recorded. The mean haemoglobin values at admission did not differ statistically between the two groups (*p* = 0.9), with 11.96 mg/dL (SD 1.76; range from 7 to 16.2) in the Pre-O group and 11.97 mg/dL (SD 1.7; range from 5 to 18.2) in the Post-O group. The day after surgery (24 h), the mean values of Hb decreased respectively to 9.43 mg/dL (SD 1.29; range from 5.7 to 13.5) and 9.56 mg/dL (SD 1.31; range from 5.6 to 14.6) (*p* = 0.21). The lowest Hb is achieved after 2.08 days (SD 2.02; range from −7 to 9) in the Pre-O group with average values of 8.33 mg/dl (SD 0.95; range from 5.1 to 13.1); while in the Post-O group is reached after 1.96 days (SD 2.14; range from −12 to 13) with average values of 8.42 mg/dL (SD 1; range from 3.5 to 13.1). However, there are no significant differences related to the day (*p* = 0.5) and the lowest Hb value reached (*p* = 0.3). There was a significant difference between the two groups in the Hb at discharge (*p* = 0.003), with 10.07 mg/dl (SD 0.82; range from 8.2 to 13.3) in the Pre-O group and 10.27 mg/dl (SD 0.84; range from 8.5 to 14.5) in the Post-O group. A total of 541 transfusions were performed in the Pre-o Group, with a mean of 1.84 (SD1.4; range from 0 to 8) transfusions per patient; 551 transfusions were performed in the Post-O group with a mean of 1.7 (SD 1.46; range from 0 to 8) transfusions per patient (*p* = 0.2). No significant difference was found in relation to LOS, pre- and post-surgical days.

**Table 2:** Hb and transfusions: Pre-O vs Post-O

	Pre-O group	Post-O group	***P*** value
**Number of patients**	296	324	
**Hb - admission** (mg/dL; SD; range)	11.96 (1.76; 7 to 16.2)	11.97 (1.70; 5 to 18.2)	0.9
**Hb 24 h post-surgery** (mg/dL; SD; range)	9.43 (1.29; 5.7 to 13.5)	9.56 (1.31; 5.6 to 14.6)	0.2
**Hb - discharge** (mg/dL; SD; range)	10.07 (0.82; 8.2 to 13.3)	10.27 (0.84; 8.5 to 14.5)	0.003 *
**Hb - lowest peak** (mean; SD; range)	8.33 (0.95; 5.1 to 13.1)	8.42 (1; 3.5 to 13.1)	0.3
**Post-surgery lowest peak day** (mean; SD; range)	2.08 (2.02; −7 to 9)	1.96 (2.14; −12 to 13)	0.5
**Total transfusions**	541	551	
**Transfusions per patient** (mean; SD; range)	1.84 (1.4; 0 to 8)	1.7 (1.46; 0 to 8)	0.2
**Length of stay** (mean; SD; range)	11.83 (4.34; 5 to 45)	12.29 (4.49; 5 to 39)	0.2
**Pre-op days** (mean; SD; range)	2.44 (2.34; 0 to 15)	2.32 (2.13; 0 to 21)	0.5
**Post-op days** (mean; SD; range)	9.38 (3.73; 0 to 43)	9.96 (4.01; 1 to 37)	0.06

*statistically significant

### Hb and transfusion: cephalomedullary nail

The data on patients who underwent cephalomedullary nailing are reported in Table 3. The Pre-O group includes 198 patients with an average age of 84.6 years (SD 6.52; range from 65 to 98); the Post-O group includes 210 patients with a mean of 84.9 years (6.4; from 66 to 99) (*p* = 0.6). The mean Hb at admission was 11.73 mg/dL (SD 1.69; range from 7 to 15.6) in the Pre-O group and 11.61 mg/dL (SD 1.66; range from 5 to 15.3) in the Post-O group (*p* = 0.5). Twenty-four hours after surgery, the Hb was 9.21 mg/dl (SD 1.28; range from 5.7 to 13.5) and 9.25 mg/dL (SD 1.17; range from 5.6 to 13.1), respectively (*p* = 0.7). Hb at discharge was 10.1 mg/dL (SD 0.82; range from 8.2 to 9.9) in the Pre-O group and 10.2 mg/dL (SD 0.8; range from 8.5 to 13.1) in the Post-O group (*p* = 0.3). In the Pre-O group, the lowest value of Hb is 1.9 days (SD 2.09; range from −7 to 9) after surgery with an average of 8.31 mg/dl (SD 0.94; range from 5.1 to 13.1); in the Post-O group, it is 1.54 days (SD 2.17; range from −12 to 13) later, with average values of 8.36 mg/dl (SD 1.02; range from 3.5 to 12.5). Again, there are no significant differences. The number of transfusions was 379 in the Pre-O group with a mean of 1.93 (SD 1.45; range from 0 to 8) transfusions per patient; 393 in the Post-O group with 1.87 (SD 1.56; range from 0 to 8) transfusions per patient (*p* = 0.7).

**Table 3:** Cephalomedullary nail

NAIL	Pre-O group	Post-O group	***P*** value
**Number of patients**	198	210	
**Age** (mean; SD; range)	84.6 (6.52; from 65 to 98)	84.9 (6.4; from 66 to 99)	0.6
**Sex**	36 M; 162 F	44 M; 166 F	
**Hb - admission** (mg/dL; SD; range)	11.73 (1.69; 7 to 15.6)	11.61 (1.66; 5 to 15.3)	0.5
**Hb 24 h post-surgery** (mg/dL; SD; range)	9.21 (1.28; 5.7 to 13.5)	9.25 (1.17; 5.6 to 13.1)	0.7
**Hb - discharge** (mg/dL; SD; range)	10.11 (0.82; 8.2 to 9.9)	10.2 (0.8; 8.5 to 13.1)	0.3
**Hb - lowest peak** (mean; SD; range)	8.31 (0.94; 5.1 to 13.1)	8.36 (1.02; 3.5 to 12.5)	0.6
**Post-surgery lowest peak day** (mean; SD; range)	1.9 (2.09; -7 to 9)	1.54 (2.17; -12 to 13)	0.09
**Total transfusions**	379	393	
**Transfusions per patient** (mean; SD; range)	1.93 (1.45; 0 to 8)	1.87 (1.56; 0 to 8)	0.7
**Length of stay** (mean; SD; range)	11.83 (4.34; 5 to 45)	12.29 (4.49; 5 to 39)	0.2
**Pre-op day** (mean; SD; range)	2.44 (2.34; 0 to 15)	2.32 (2.13; 0 to 21)	0.5
**Post-op day** (mean; SD; range)	9.38 (3.73; 0 to 43)	9.96 (4.01; 1 to 37)	0.06

### Hb and transfusions: HHA

The data related to patients who underwent hemiarthroplasty (HHA) are reported in Table 4. The Pre-O group includes 90 patients with a mean age of 83.8 years (SD 7.2; range from 65 to 96); the Post-O group included 97 patients with a mean age of 82.5 years (SD 7.96; range from 55 to 99) (*p* = 0.2). Hb at admission was 12.36 mg/dL (SD 1.85; range from 8 to 16.2) in the Pre-O group and 12.61 mg/dL (SD 1.56; range from 8.8 to 18.2) in the Post-O group (*p* = 0.31). Twenty-four hours after surgery, the Hb did not differ significantly between the two groups (*p* = 0.1). In these patients, the Hb at discharge differs significantly in the two groups (*p* = 0.0001), 9.96 mg/dL (SD 0.82; range from 8.6 to 9.4) in the Pre-O group and 10.43 mg/dL (SD 0.8; range from 8.9 to 12.9) in the Post-O group. In the Pre-O group, the lowest peak of Hb is 8.39 mg/dl (SD 0.97; range from 6 to 11.5), reached 2.43 days (SD 1.84; range from −7 to 9) after surgery, while in the Post-O group is 8.62 mg/dl (SD 0.9; range from 6.5 to 10.9), 2.71 days (SD 1.92; range from −5 to 11) after surgery. A further significant difference is the average number of transfusions per patient (*p* = 0.03). Indeed, 153 transfusions were performed in the Pre-O group with an average of 1.7 (SD 1.31; range from 0 to 6) transfusions per patient; in the Post-O group 127 transfusions were performed with an average of 1.31 (SD 1.13; range from 0 to 6) transfusions per patient. In these patients, the LOS and the pre-surgery days do not differ, while in the Pre-O group, the time between surgery and discharge is significantly reduced (*p* = 0.008).

**Table 4:** HHA

HHA	Pre-O group	Post-O group	***P*** value
**Number of patients**	90	97	
**Age** (mean; SD; range)	83.8 (7.2; from 65 to 96)	82.5 (7.96; from 55 to 99)	0.2
**Sex**	33 M; 57 F	20 M; 77 F	
**Hb - admission** (mg/dL; SD; range)	12.36 (1.85; 8 to 16.2)	12.61 (1.56; 8.8 to 18.2)	0.3
**Hb 24 h post-surgery** (mg/dL; SD; range)	9.84 (1.21; 7.2 to 13.2)	10.13 (1.37; 7.4 to 14.6)	0.1
**Hb - discharge** (mg/dL; SD; range)	9.96 (0.82; 8.6 to 9.4)	10.43 (0.8; 8.9 to 12.9)	0.0001*
**Hb - lowest peak** (mean; SD; range)	8.39 (0.97; 6 to 11.5)	8.62 (0.9; 6.5 to 10.9)	0.09
**Post-surgery lowest peak day** (mean; SD; range)	2.43 (1.84; −7 to 9)	2.71 (1.92; −5 to 11)	0.3
**Total transfusions**	153	127	
**Transfusions per patient** (mean; SD; range)	1.7 (1.31; 0 to 6)	1.31 (1.13; 0 to 6)	0.03*
**Length of stay** (mean; SD; range)	11.78 (3.93; 7 to 25)	12.77 (5.03; 6 to 39)	0.1
**Pre-op day** (mean; SD; range)	3.02 (2.78; 0 to 15)	2.49 (2.25; 0 to 16)	0.2
**Post-op day** (mean; SD; range)	8.75 (2.72; 4 to 17)	10.27(4.66; 1 to 37)	0.008*

*statistically significant

### Hb and transfusion: THA

In patients undergoing THA, there are no significant differences between the two groups (Table 5). The Pre-O group includes 8 patients with a mean age of 74 years (SD 7.38; range from 63 to 87); the Post-O group comprises 17 patients with an average age of 72 years (SD 9.5; range from 53 to 93) (*p* = 0.6). Hb on admission was 13.52 mg/dL (SD 0.94; range from 11.5 to 14.7) in the Pre-O group; 12.94 mg/dL (SD 1.68; range from 10.3 to 15.8) in the Post-O group (*p* = 0.37). Twenty-four hours after THA, Hb was 10.62 mg/dL (SD 0.61; range from 10 to 11.6) and 10.16 mg/dL (SD 1.35; range from 7.8 to 12.6), respectively (*p* = 0.4). On discharge, the values were 10.23 mg/dl (SD 0.68; range from 9.5 to 11.8) for the Pre-O group and 10.36 mg/dl (SD 1.37; range from 8.7 to 14.5) with for the Post-O group. The lowest level of Hb was 3.17 days (SD 1.6; range from 2 to 6) after surgery, with 8.57 mg/dL (SD 0.58; range from 7.7 to 9.3) in the Pre-O group; and after 2.87 days (SD 1.75; range from 0 to 11) with 8.1 mg/dL (SD 0.86; range from 6.4 to 9.7) in the Post-O group (*p* = 0.7). The patients of the Pre-O group received a total of 9 transfusions, 1.12 (SD 0.64; range from 0 to 2) transfusions per patient; 31 transfusion were performed in the Post-O group, 1.82 (SD 1.42; range from 0 to 5) transfusions per patient (*p* = 0.12).

**Table 5:** THA

THA	Pre-O group	Post-O group	***P*** value
**Number of patients**	8	17	
**Age** (mean; SD; range)	74 (7.38; from 63 to 87)	72 (9.5; from 53 to 93)	0.6
**Sex**	2 M; 6 F	6 M; 11 F	
**Hb - admission** (mg/dL; SD; range)	13.52 (0.94; 11.5 to 14.7)	12.94 (1.68; 10.3 to 15.8)	0.4
**Hb 24 h post-surgery** (mg/dL; SD; range)	10.62 (0.61; 10 to 11.6)	10.16 (1.35; 7.8 to 12.6)	0.4
**Hb - discharge** (mg/dL; SD; range)	10.23 (0.68; 9.5 to 11.8)	10.36 (1.37; 8.7 to 14.5)	0.8
**Hb - lowest peak** (mean; SD; range)	8.57 (0.58; 7.7 to 9.3)	8.1 (0.86; 6.4 to 9.7)	0.2
**Post-surgery lowest peak day** (mean; SD; range)	3.17 (1.6; 2 to 6)	2.87 (1.75; 0 to 11)	0.7
**Total transfusions**	9	31	
**Transfusions per patient** (mean; SD; range)	1.12 (0.64; 0 to 2)	1.82 (1.42; 0 to 5)	0.2
**Length of stay** (mean; SD; range)	10.71 (2.42; 9 to 16)	13.76 (6.8; 7 to 34)	0.2
**Pre-op day** (mean; SD; range)	1.42 (0.53; 1 to 2)	2.64 (1.65; 1 to 7)	0.06
**Post-op day** (mean; SD; range)	9.28 (2.28; 7 to 14)	11.12 (6.2; 5 to 27)	0.4

## Discussion

A significant health care burden is related to the management of elderly patients with hip fractures [31–33]. Indeed, the incidence of hip fractures, as well as the incidence of all age-related fractures, is progressively increasing given the increase of life expectancy [31, 33–35]. Treating the fracture is not enough, and a comprehensive management of the patient is needed. Multiple comorbidities in the elderly population are extremely common [36, 37]. Consequently, these patients take multi-therapies that need to be considered before administering further drugs [36, 37].

In elderly patients with a hip fracture, it is important to monitor blood loss; they are often anaemic, regardless of the fracture [38]. Anaemia may delay surgical treatment, resulting in further blood loss. In these patients, transfusions are needed. However, the threshold value of Hb to begin the transfusion is debated. Some studies report that, in such patients, transfusions are associated with an increased risk of post-surgical complications and long-term mortality [39]; others report a decreased rate of delirium and a faster post-surgical functional recovery [40–42]. In others still, no association between the number of transfusions and the risk of mortality is recognised [43]. Some studies proposed a threshold value of Hb < 7 mg/dL as a trigger for transfusions in hemodynamically stable patients [44]. However, elderly patients with hip fractures often have different comorbidities, especially cardiovascular; in addition, it must also be considered the age-related fragility of these patients, in whom the fracture can lead to rapid hemodynamic imbalance even if they may be initially stable. In the Orthopaedic Department of the San Giovanni di Dio e Ruggi d’Aragona Hospital of Salerno, patients are monitored every day to maintain Hb to adequate levels. Since February 1, 2018, an orthogeriatrician works in the Orthopaedic Department, to promote functional recovery, reducing pre- and post-surgical complications and both short- and long-term mortality of the patients, thus reducing LOS, re-hospitalisations and related costs [45, 46]. The present study assessed whether the presence of an orthogeriatrician affects the blood management and the number of transfusions in patients with hip fractures. Regardless of the surgical treatment performed, there are no significant differences related to Hb at admission and 24 h after surgery, and the number of transfusions per patient does not differ significantly despite the involvement of the orthogeriatrician.

However, Hb at discharge was significantly higher in the Post-O group (*p* = 0.003). This difference probably results from a more comprehensive management provided by the orthogeriatrician, which also allows a faster recovery of the values of the Hb. Analysing the data according to the surgical procedure performed, there are some differences. Indeed, there are no differences related to Hb levels and the number of transfusions in the patients treated with an intramedullary nail and with THA. Differently, in the patients undergoing HHA, Hb at discharge was significantly higher in the patients managed by the orthogeriatrician (*p* = 0.0001); in addition, in these patients, the number of transfusions per patient was reduced (*p* = 0.03), although the post-surgical LOS was increased (*p* = 0.008). Despite the lack of significant differences in the number of transfusions performed, the higher Hb value at discharge may have a significant impact. Low levels of Hb can increase the LOS and thus hospital-related costs. In addition, although the risk of morbidity and mortality may not be affected, higher values of Hb at discharge reduce the risk of re-admission [47]. This allows to prevent further costs related to both the hospitalisation and the need for transfusions in case of hemodynamic imbalance of patients, in particular those with associated cardiovascular diseases.

The strength of this study is related to a large number of patients included. The demographic characteristics of patients and comorbidities do not differ significantly, except for patients with heart failure, who presented less often in the Post-O period (*p* = 0.007). In addition, the surgical procedures were performed in both groups by the same surgical team; therefore, the experience of surgeons should not affect the outcomes. The limitations are related to the retrospective nature of the study. The included patients were at least ASA III, as assessed by a consultant anaesthetist with a special interest in hip fracture management in the elderly. The computerised data collection and retrieval system in our health care system does not allow us to dwell further in this field; this would have made it possible to define more precisely the work of the orthogeriatrician. Furthermore, no data are available on the patient after discharge, so it is not possible to assess the long-term outcomes of the two groups of patients.

## Conclusion

The introduction of the orthogeriatrician in an orthopaedic ward in which elderly patients are treated for hip fracture does not significantly change the number of transfusions per patient performed during the LOS. However, it allows to discharge the patients with significantly higher Hb values. In patients undergoing HHA, in addition to the highest Hb at discharge, the number of average transfusions per patient is significantly reduced. The increased values of Hb at discharge and the decreased number of transfusions in patients undergoing HHA indicate the involvement of an orthogeriatrician in the management of elderly patients with hip fractures. Indeed, with a higher Hb at discharge, the risk of anemisation of patients is reduced; consequently, also the costs related to possible re-admission and further transfusions are reduced.

## Data Availability

This study does not contain any third materials.

## Abbreviations

HHA: Hip hemiarthroplasty
THA: Total hip arthroplasty
LOS: Length of hospital stay
Hb: Haemoglobin
